# Expanding the miRNA Transcriptome of Human Kidney and Renal Cell Carcinoma

**DOI:** 10.1155/2018/6972397

**Published:** 2018-07-03

**Authors:** Adam P. Sage, Brenda C. Minatel, Erin A. Marshall, Victor D. Martinez, Greg L. Stewart, Katey S. S. Enfield, Wan L. Lam

**Affiliations:** Department of Integrative Oncology, British Columbia Cancer Research Centre, Vancouver, BC, Canada

## Abstract

Despite advancements in therapeutic strategies, diagnostic and prognostic molecular markers of kidney cancer remain scarce, particularly in patients who do not harbour well-defined driver mutations. Recent evidence suggests that a large proportion of the human noncoding transcriptome has escaped detection in early genomic explorations. Here, we undertake a large-scale analysis of small RNA-sequencing data from both clear cell renal cell carcinoma (ccRCC) and nonmalignant samples to generate a robust set of miRNAs that remain unannotated in kidney tissues. We find that these novel kidney miRNAs are also expressed in renal cancer cell lines. Moreover, these sequences are differentially expressed between ccRCC and matched nonmalignant tissues, implicating their involvement in ccRCC biology and potential utility as tumour-specific markers of disease. Indeed, we find some of these miRNAs to be significantly associated with patient survival. Finally, target prediction and subsequent pathway analysis reveals that miRNAs previously unannotated in kidney tissues may target genes involved in ccRCC tumourigenesis and disease biology. Taken together, our results represent a new resource for the study of kidney cancer and underscore the need to characterize the unexplored areas of the transcriptome.

## 1. Introduction

Despite recent advancements in the diagnosis and treatment of kidney cancer, patients are faced with a poor prognosis, especially when diagnosed at a later stage [1]. Kidney cancer is a heterogeneous disease with multiple subtypes, of which clear cell renal cell carcinoma (ccRCC) is the most frequently observed, accounting for 70–75% of cases [2]. While environmental risk factors including hypertension, smoking, obesity, and a history of chronic kidney disease may modulate an individual's susceptibility, ccRCC arises from molecular aberrations that can be both sporadic and inherited [2, 3]. Many of these alterations result from DNA copy number losses, mutations, and hypermethylation events, commonly affecting genes associated with cellular metabolism [4, 5]. The most frequently affected gene is the *von Hippel-Lindau* (VHL) tumour suppressor gene, while other molecular disruptions affecting multiple components of the PI3K-mTOR and AMPK signaling pathways have also been described [5, 6]. Considering the close association of metabolic reprogramming with ccRCC development and progression, remarkable advances have been made in the treatment of ccRCC patients with the use of antiangiogenic therapies [7]. Despite the increased treatment efficacy of antiangiogenic therapies, patient outcome is impaired by the lack of clinically relevant diagnostic or prognostic markers [8, 9].

The increased availability of next-generation sequencing has led to a dramatic increase in the understanding of noncoding RNAs (ncRNAs). Perhaps, the most well-studied type of ncRNA is microRNAs (miRNAs), short (~22 nt) transcripts that have emerged as critical regulators of gene expression. Since the discovery of *lin-4* in 1993, miRNAs have been found to regulate a multitude of transcripts and their subsequent cellular processes, including proliferation, metabolism, apoptosis, and development [10–12]. Moreover, the relatively long half-life of miRNAs makes them attractive candidates for biomarkers of disease [13]. miRNAs have been observed to be critical to kidney development, physiology, and pathology. For instance, a cluster of miRNAs (miR-17~92) has been shown to regulate nephrogenesis; both miR-9 and miR-374 are observed to suppress claudin-14 and affect Ca^2+^ readsorption in the ascending limb of Henle; and recent studies have detected aberrant expression of miRNAs in kidney tumours [13–16]. However, despite the mounting evidence of a role for miRNAs in ccRCC, they have yet to be used in kidney cancer diagnostics.

miRNAs have been described to be genus- and tissue-specific; yet, initial characterizations of the human miRNA transcriptome have relied heavily on sequence abundance and conservation, while using relatively low-depth coverage techniques. In light of this, it has been hypothesized that the human genome likely encodes a markedly greater number of miRNAs than currently annotated, which may be able to be identified through a focus on individual tissues and cell lineages [17]. Indeed, recent genome-wide studies have uncovered miRNAs that have previously escaped detection and have observed that these newly detected miRNAs are highly tissue-specific [18]. Additionally, previously uncharacterized miRNAs may in fact represent novel regulators of tissue-specific biology and pathogenesis and may have utility in the clinic as disease markers. Thus, in this study, we use a large-scale analysis of high-throughput sequencing data to probe for novel miRNAs in human kidney tissue. Discovery of these previously unannotated miRNAs provides a new resource to delineate ccRCC pathogenesis.

## 2. Materials and Methods

### 2.1. Small RNA-Sequencing and Data Collection

A cohort of clear cell renal cell carcinoma (ccRCC) tumours with paired nonmalignant tissues (*n* = 71), as well as unpaired tumours (*n* = 502), was processed by The Cancer Genome Atlas (TCGA) Research Network (http://cancergenome.nih.gov/). Small RNA-sequencing data were generated on the Illumina HiSeq200 platform and were acquired from the Cancer Genomics Hub (cgHUB) Data Repository (dbgap Project ID: 6208) under the TCGA-KIRC data collection heading. All data analyzed in this experiment are available publically.

### 2.2. Preprocessing of Small RNA-Sequencing Data

All raw sequence data obtained from TCGA were processed using a previously published custom sequence analysis pipeline designed for small RNA sequence detection [19]. Raw BAM files from TCGA were first converted to FASTQ files of unaligned reads. Unaligned reads were trimmed based on their Phred quality score, which is required to be ≥20. The trimmed reads were then realigned to the current build of the human genome (hg38 annotation) using the Spliced Transcripts Alignment to a Reference (STAR) aligner.

### 2.3. Detection and Filtering of Novel miRNA Sequences

Through the OASIS online small-RNA-sequencing analysis platform, novel miRNA sequences were predicted using the miRDeep2 algorithm [20]. miRDeep2 takes both relative free energy and the *p* values associated with random folding to predict species with miRNA-like structure and to generate a miRDeep2 score reflective of the reliability of the prediction. To confirm the validity of these predictions, stringent manual assessments were performed to generate a robust set of previously unannotated miRNA sequences. Manual filtering was based on (1) an adequate number of sequencing reads covering each locus (≥10 reads); (2) no presence of rRNA/tRNA reads, based on the Rfam database [21]; (3) significant (*p* < 0.05) probability of miRNA-like secondary structure; and (4) removal of duplicate sequences. Standard nucleotide blast (BLASTn) was performed on all remaining predicted novel miRNA sequences using the BLAST+ command line application [22]. This step ensures that any sequences with homology to miRNAs annotated in miRBase v21 are not included in the final list of predicted sequences. Sequences with an expect (*E*) value of <0.1 were considered previously annotated and discarded from further analyses. The mean and standard deviation of the GC content of all predicted novel miRNA sequences were calculated in order to remove any transcripts with GC content ± 2 standard deviations from the mean. Expression levels of these newly detected sequences were assessed on a per-sample basis using the algorithm featureCounts v1.4.6 [23–25]. Quantification data were normalized according to the weighted trimmed mean of the log expression ratios (trimmed mean of *M* values, TMM method). Previously unannotated miRNA species were considered to be expressed if the sum of the sequencing reads across all samples was at least 10 reads. Finally, sequences were queried for their presence in five previous studies that have identified novel miRNAs in various tissues [17, 18, 26–28] (Supplemental Table 1).

### 2.4. Validation of miRNA Expression in Cell Lines

We have performed an in-depth analysis of the small noncoding RNA transcriptome of the National Cancer Institute's cancer cell line panel (NCI-60), which includes eight renal cancer cell lines (A498, CAKI-1, 786-0, TK-10, UO-31, ACHN, RXF393, and SN12C) [28]. Small RNA-sequencing data generated from these renal cancer cell lines were processed as described previously to detect and quantify the expression of the predicted novel miRNA sequences discovered in ccRCC tumours. Cell line characteristics and detailed sequencing information are available in Supplemental Table 2. An expression cutoff (TMM > 0.1) was used to ensure that the sequencing reads were expressed in at least one of the renal cancer cell lines. Predicted miRNA sequences that were expressed in data obtained from both TCGA patient samples and NCI-60 cell lines were considered to be validated and were assigned a unique ID consisting of Knm (kidney novel miRNA sequence), followed by the locus position (i.e., Knm22_2209).

### 2.5. Differential Expression of Previously Unannotated miRNAs in ccRCC and Nonmalignant Tissue

As performed in the cell line validation step, an expression cutoff of TMM > 0.1 in 10% of samples was used to determine newly detected miRNA loci that had detectable expression in both ccRCC tumour samples and paired nonmalignant tissue (*n* = 71). Fold change values were calculated as the ratio of expression of the newly detected miRNA loci in tumours to their expression in nonmalignant tissue. A Student *t*-test was performed on the TMM expression values of the newly detected miRNAs in RStudio v3.3.3 to test for statistically significant differences in expression between ccRCC tumours and paired nonmalignant samples. Multiple correction analyses were performed using the Benjamini-Hochberg (BH) correction, to account for the large number of samples and probes being analyzed. Additionally, unsupervised hierarchical clustering analysis using average distance and Pearson correlation metrics of differentially expressed miRNAs was performed to visualize their expression on a per-sample basis (Supplemental Figure 2).

#### 2.5.1. Cell Culture and Real-Time Quantitative PCR (RT-qPCR)

The renal cancer cell line TK-10 and the immortalized nonmalignant embryonic kidney cell line HEK-293T were used to further explore the expression of the previously unannotated miRNAs and their deregulation in ccRCC tumours in vitro. TK-10 cells were cultured in RPMI 1640 + 10% FBS, while HEK-293T were cultured in DMEM + 10% FBS. Both cell lines were maintained in an incubator at 37°C and 5% CO_2_. Once confluent, cells were harvested for RNA extraction using the Quick-RNA™ MiniPrep Kit (Zymo Research, Catalog number R1055), following manufacturer's guidelines. Custom reverse-transcription primers specific to the mature miRNA sequence were obtained for the novel miRNA candidates Knm3_1968 (GCAGAUUCCCAGAGUGGGACAG) and Knm17_1130 (UGAGGUGGAGGGUUGUGGGA) using the Custom TaqMan® Small RNA Assay Design Tool from Thermo Fisher. The cDNA conversions were performed with the TaqMan MicroRNA Reverse Transcription Kit according to manufacturer's instructions using 2 ng/*μ*L RNA samples for both cell lines. Finally, RT-qPCR analyses using the custom primers generated from Thermo Fisher were performed in triplicate in an Applied Biosystems® 7500 Real-Time PCR System. Relative miRNA expression was calculated via the 2^−ΔΔCt^ method and normalized to the expression of U6 snRNA.

### 2.6. Survival Analyses of Previously Unannotated miRNAs

Phenotypic information for all ccRCC tumour samples was obtained from GDC (TCGA-KIRC) through UCSC Xena (http://xena.ucsc.edu/). Samples were sorted by high to low miRNA expression, and tertiles were defined. Patients were categorized by vital status and days to death/last follow-up. The Gehan-Breslow-Wilcoxon test was used to assess the significance of the associations between miRNA expression and patient outcome for each miRNA sequence examined. The log-rank test was also considered.

### 2.7. Protein-Coding Gene Target Prediction and Pathway Enrichment Analysis

To determine the potential target genes of the newly detected kidney miRNAs, they were queried against all human 3′ untranslated region (UTR) sequences, acquired from Ensembl through the BioMart tool (https://www.ensembl.org), using the miRanda v3.3a algorithm [29]. Predicted gene targets of the miRNAs were validated by individually running five separate scrambled sequences through the algorithm. Predicted targets that overlapped between any of the scrambled sequences and the true sequence were discarded. Strict parameters were used in the target prediction analysis, specifically an alignment score of ≥140 and an energy threshold of ≤−20 kcal/mol (Supplemental Table 3). Gene symbols identified by the miRanda algorithm and predicted to be targeted by at least 10% of the previously unannotated miRNAs were submitted to a comprehensive pathway enrichment analysis using pathDIP v.2.5.21.6, which assesses enrichment of the target genes in pathways obtained from 15 distinct public pathway resources (literature-curated (core) pathway memberships) [30]. In this study, we report all pathways enriched with corrected *p* values ≤ 0.05.

## 3. Results

### 3.1. Discovery of Previously Unannotated miRNAs in ccRCC and Normal Kidney Tissue

**Figure 1:**
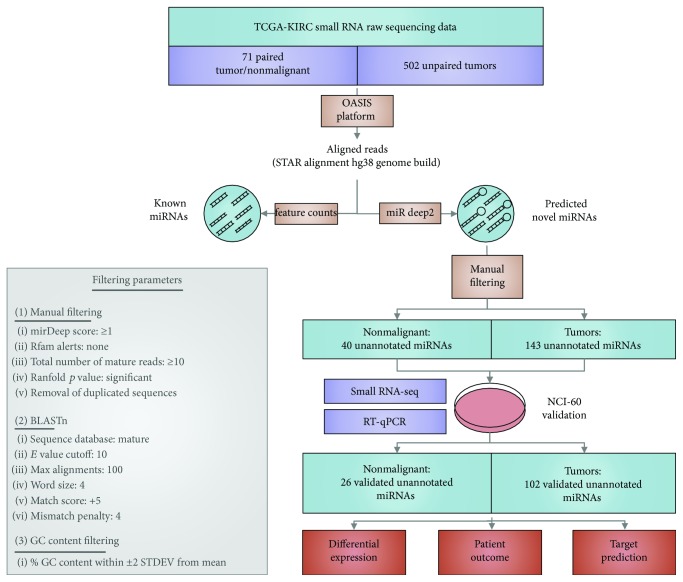
Experimental analysis pipeline. Detailed diagram of the analyses used to generate predicted and validated miRNA sequences previously unannotated in kidney tissues, along with subsequent explorations into their biological relevance.

Small RNA-sequencing data of ccRCC tumours (unpaired (*n* = 502) and tumours with matched nonmalignant tissue (*n* = 71)) were obtained from cgHUB and processed, and data were analyzed using the miRDeep2 prediction algorithm through the OASIS platform [20, 23]. The raw output of this analysis predicted 96 and 280 unique, previously unannotated miRNAs in nonmalignant tissue and ccRCC tumours, respectively. Manual filtering based on read quality, likelihood of miRNA-like secondary structure, and significant folding values, followed by probing the degree of similarity with known miRNAs using the BLASTn algorithm, and removal of sequences with aberrant GC content, resulted in 40 nonmalignant and 143 tumour previously unannotated miRNAs (Figure 1, Supplemental Table 1).

To validate the occurrence of the previously unannotated miRNAs in kidney tissues, we assessed their expression in small RNA-sequencing data generated from the NCI-60 cell line panel [28]. A miRNA was considered to be expressed in the eight renal cell lines included in the panel if it had a normalized expression value of greater than 0.1 in at least one of the renal cancer cell lines. In these cell lines, 26 (65%) novel miRNAs detected in nonmalignant samples and 102 (71%) miRNAs detected in ccRCC tumours were also expressed, strengthening their confidence as true miRNA sequences in human renal tissues and suggesting possible relevance to kidney function and pathology. Thus, we sought to further examine the role that these unannotated and validated miRNAs expressed in kidney tissues may have in ccRCC tumourigenesis and their potential clinical relevance.

### 3.2. Previously Unannotated miRNAs Are Deregulated in ccRCC Tumours

Recent evidence suggests that widespread disruption of miRNA-coding gene regulatory networks is common in many cancer types. Perturbation of coding gene expression by miRNA-based regulation can be achieved in tumours through loss of a tumour-suppressive miRNA or the gain of an oncogenic miRNA. Thus, we analyzed the expression of these previously unannotated miRNA sequences in tumours and paired nonmalignant tissue to identify potentially novel oncogenic and tumour-suppressive miRNAs in ccRCC.

**Figure 2:**
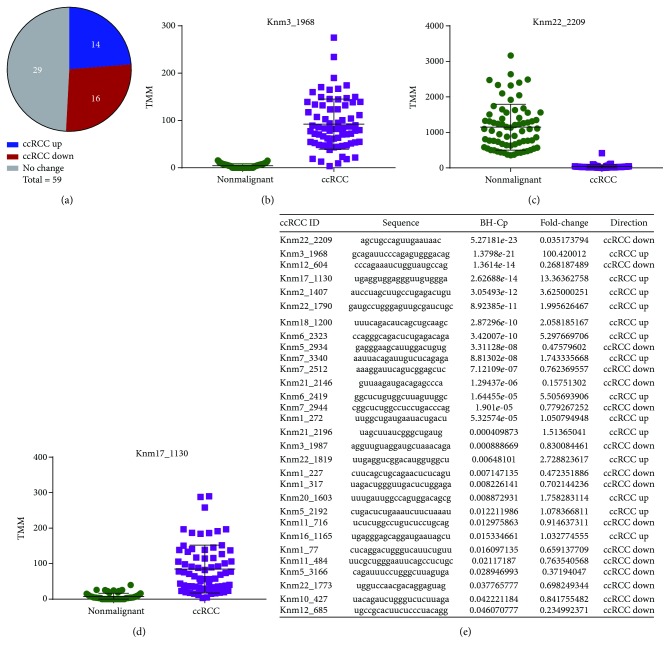
Differential expression of previously unannotated miRNAs between nonmalignant and ccRCC samples. (a) Pie chart representing the proportion of miRNAs that are significantly differentially expressed between the samples. (b) Normalized expression of the previously unannotated miRNA Knm3_1968 in individual nonmalignant (green) and ccRCC samples (purple). (c) Normalized expression of Knm22_2209 in individual nonmalignant (green) and ccRCC samples (purple). (d) Normalized expression of Knm17_1130 in individual nonmalignant (green) and ccRCC samples (purple). (e) Summary of differential expression results for each previously unannotated miRNA. BHC-p represents the corrected *p* value for the differential expression calculated using Student's *t*-test and Benjamini-Hochberg multiple correction analysis.

After filtering for expression in both groups of samples, 59 previously unannotated miRNA loci were considered to be expressed in both ccRCC tumours and nonmalignant samples. A Student's *t*-test analysis revealed 30 of these 59 miRNAs to be significantly differentially expressed between the two groups (BH-*p* < 0.05). An analysis of the average fold change values between paired samples revealed that 14 miRNAs were significantly upregulated in ccRCC tumours, while the other 16 were downregulated (Figure 2). A subset of these miRNAs is of particular interest due to the magnitude of their expression differences between tumour and nonmalignant samples (Figure 2; Supplemental Figure 2). For example, the previously unannotated miRNA Knm22_2209 has an almost complete loss of expression in ccRCC tumours (28-fold downregulated in tumours, BH-*p* = 5.3 × 10^−23^), while previously unannotated miRNAs Knm3_1968 and Knm17_1130 show a 100- and 13-fold increase in expression in ccRCC samples, respectively (BH-*p* = 1.4 × 10^−21^, 2.6 × 10^−14^). These findings serve to not only highlight the potential role of currently unannotated miRNAs in kidney cancer but also warrant investigation into their uses as biological markers of cancer onset.

### 3.3. Patient Outcome Predicted by Previously Unannotated miRNAs

**Figure 3:**
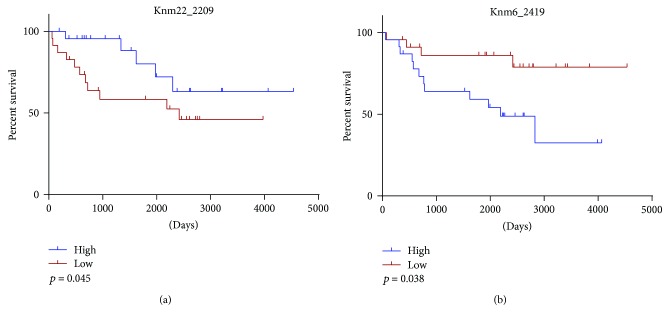
ccRCC patient overall survival predicted by previously unannotated miRNAs. Red lines represent patients with expression of the miRNA in the lower tertile of expression, while blue lines represent the upper tertile of expression. Calculation of *p* values for Knm22_2209 (a) and Knm6_2419 (b) was performed using the Gehan-Breslow-Wilcoxon test.

The differential expression of these unannotated miRNAs in paired ccRCC tumour samples suggests their potential roles in kidney cancer and unexplored clinical utility. As such, we sought to examine whether any of the unannotated miRNA sequences deregulated in ccRCC tumours were associated with patient outcome. We examined differences in patient survival in those with high expression of a miRNA to those with low expression, defined by tertiles. Survival analysis was performed using the Gehan-Breslow-Wilcoxon test on phenotype data obtained from UCSC Xena and the expression profiles of the unannotated miRNA loci. Interestingly, two of the significantly differentially expressed unannotated miRNAs in paired ccRCC tumour samples displayed striking associations with patient survival (Figure 3). Again, the unannotated miRNA Knm22_2209 (28-fold downregulated in ccRCC, BH-*p* = 5.3 × 10^−23^) is particularly noteworthy due to the clear correlation between its low expression and poor overall survival (Figure 3(a), *p* = 0.045). Alternatively, the Knm6_2419 miRNA is significantly upregulated in ccRCC tumours (FC = 5.5, BH-*p* = 1.6 × 10^−5^), and its high expression is significantly associated with a worsened overall patient survival (Figure 3(b), *p* = 0.038). Taken together, these results emphasize the clinical potential of previously undetected miRNAs.

### 3.4. Genes and Pathways Targeted by Newly Detected miRNAs

In order to gain deeper insight into the biological relevance of the newly detected miRNAs, target prediction analysis was performed using the miRanda v3.3a algorithm on the set of 30 miRNAs significantly differentially expressed in ccRCC. This algorithm reports potential gene targets for the analyzed sequences based on sequence complementarity and the thermodynamic stability of the complementary RNA duplexes [29]. Predicted target genes with an alignment score ≥ 140 and an energy threshold of ≤−20 kcal/mol were considered as potential gene targets of our newly detected miRNAs in ccRCC. To generate a broad list of possible gene targets and their associated pathways that may be affected by the regulatory action of the 30 miRNAs, we examined genes that were targeted by at least 10% of these miRNAs (Supplementary Table 3), which were subsequently analyzed for pathway enrichment using pathDIP [30].

**Figure 4:**
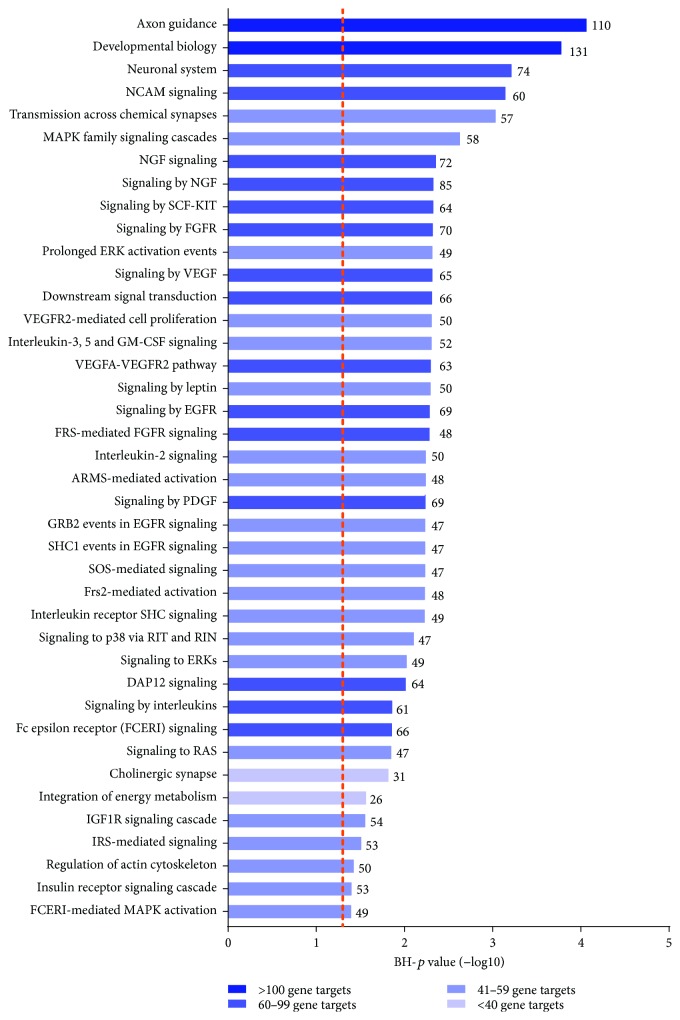
Pathways significantly enriched in protein-coding gene targets of previously unannotated miRNAs. Pathways are plotted according to the significance value associated with their enrichment in predicted miRNA gene targets. Individual genes in each pathway are predicted to be targeted by at least 10% of the previously unannotated miRNAs. Bars are coloured by the number of predicted gene targets involved in the pathway, with the total number of gene targets displayed on top of each bar. The orange dashed line represents the significance cutoff of BH-*p* = 0.05.

Pathway enrichment analysis revealed 63 significantly enriched core pathways (BH-*p* ≤ 0.05). Interestingly, many of the enriched pathways are associated with cellular response to extracellular stimuli, organ development, and pathways indirectly associated with cellular metabolism, including the axon guidance pathway (110 gene targets), MAPK signaling cascade (58 gene targets), signaling mediated by FGFR and EGFR (70 and 69 gene targets, resp.), as well as the VEGF pathway (65 gene targets), and insulin-mediated signaling (63 gene targets) (Figure 4) [31, 32]. Considering that kidney cancers have been recognized to associate with altered hypoxic signaling, as well as cellular metabolism [4, 33], our results suggest that these previously unannotated miRNAs may play key roles in the regulation of ccRCC development and progression.

### 3.5. In Vitro Validation of Previously Unannotated miRNAs Deregulated in ccRCC

In order to experimentally confirm the existence of these transcripts and their consequent deregulation in tumour tissues, we performed RT-qPCR using custom primers specific to Knm3_1968 and Knm17_1130, previously unannotated miRNAs strongly overexpressed in ccRCC samples. We used the TK-10 cell line, which is representative of the ccRCC subtype of renal cancer, as well as HEK-293T, which is an immortalized nonmalignant embryonic kidney cell line commonly used as a control cell line to represent nonmalignant kidney tissue. RT-qPCR results were consistent with expression data from RNA-sequencing, wherein both Knm3_1968 and Knm17_1130 were found to be overexpressed in TK-10 cells relative to HEK-293T cells (average RQ = 8.56; 16.54, resp.; Supplemental Figure 3).

## 4. Discussion

In this study, we discovered miRNAs previously unannotated in kidney tissues using the miRDeep2 algorithm which represents an increase of 11.5% from the current number of kidney-related miRNAs annotated in miRBase v21 [24]. Collectively, our findings underscore the need to accurately define the landscape of miRNA transcription in human tissues, particularly due to their emerging roles in cell biology and disease.

The miRDeep2 algorithm integrates artifacts of Dicer-based miRNA processing to generate a score for each predicted miRNA that is reflective of its likelihood of being a true positive [22]. Thus, we further processed the predicted sequences to eliminate sequences that (i) have low true positive detection rates (miRDeep2 score), (ii) share sequencing reads with other ncRNA species (tRNA/rRNA), (iii) do not meet the threshold of significance (ranfold *p* value), (iv) strongly differ from known miRNAs in G/C content, and (v) are already found in miRBase v21 (BLASTn). Together, these filtering steps enable us to generate a robust set of previously unannotated miRNAs that have low false-discovery rates.

To further validate these miRNAs in kidney tissues, we probed their expression in the NCI-60 panel of cell lines. Although the eight renal cancer cell lines do not reflect the spectrum of ccRCC in patients, they offer the opportunity to assess whether a fraction of the newly identified miRNAs occur in samples independent of TCGA. We found 128 miRNAs newly detected in kidney samples to be expressed in the eight renal cancer cell lines found in the panel. These findings, in tandem with our previous work characterizing the ncRNA transcriptome of the panel [28], serve to confirm the presence of the miRNAs detected in kidney tissues in our analysis and more broadly emphasize the extent of miRNA transcription that has previously escaped detection. Moreover, this highlights the utility of the NCI-60 cell line panel in future explorations of novel miRNA discovery and biology.

After validating their expression in cell lines, we examined the relevance of our panel of miRNAs newly detected in kidney tissues to ccRCC tumourigenesis and disease biology. Strikingly, we found a large proportion of these miRNAs to be significantly differentially expressed between ccRCC tumours and matched nonmalignant tissue. We further observed strongly deregulated miRNA sequences, both up- and downregulated in ccRCC samples. These cases—particularly Knm22_2209, Knm17_1130, and Knm3_1968—suggest that the manipulation of miRNA expression may be a factor in the tumourigenesis of ccRCC tumours. In addition, there are a number of our previously unannotated sequences that were not included in the differential expression analyses as their expression is only detected in either the nonmalignant or ccRCC paired samples. However, the expression of these sequences may be specific to either nonmalignant or cancerous kidney tissue (Supplemental Figure 2), suggesting that these miRNAs may represent exciting candidates for markers of ccRCC development or as therapeutic targets that may display limited off-target effects in normal cells.

The clinical applicability of a subset of the previously unannotated miRNAs is highlighted by our observations that several of the differentially expressed miRNAs are significantly associated with patient outcome. Specifically, the miRNAs Knm22_2209 and Knm6_2419 display significant relationships with the poor outcome of ccRCC patients. While preliminarily, these results highlight the potential impact that novel miRNA discovery can have on both cancer biology and clinical cancer intervention, warranting further characterization of the clinical relevance of previously undetected miRNAs in various pathologies.

The miRNAs discovered in our analyses are observed to target genes critical to both normal and diseased kidney biology. The axon guidance pathway mediates neuronal migration and positioning; in the kidney, this pathway has been shown to play key roles in organ development, in which deletion of critical pathway genes has been shown to lead to after-birth death due to kidney abnormalities [34]. Interestingly, 110 genes in this pathway are each targeted by at least 3 of the previously unannotated miRNAs. Studies have also implicated aberrations in genes from the axon guidance pathway with malignant cell growth and angiogenesis [35]. In fact, kidney cancers have been reported to rely on extensive metabolic reprogramming, in which most of the identified molecular drivers have been shown to participate in pathways related to cellular energy, nutrient metabolism, and oxygen sensing [5, 7]. The newly detected miRNAs in the kidney were found to target several components of the vascular endothelial growth factor (VEGF) pathway, a major regulator of angiogenesis, as well as signaling cascades in response to extracellular stimuli, such as the insulin receptor signaling cascades and FGRF and EGFR signaling.

Specifically, Knm3_1968 is found to target genes such as ACACA, which encodes acetyl-CoA carboxylase alpha, involved in fatty acid synthesis. Additionally, Knm22_2209 targets genes such as RASGRP2, which is associated with the MAPK signaling pathway. Thus, the observation that key pathways in ccRCC tumourigenesis are predicted to be targeted by the miRNAs discovered in our study, particularly axon guidance, metabolic reprogramming, and angiogenesis, emphasizes the potential regulatory role of these transcripts in kidney cancer biology.

Finally, both Knm3_1968 and Knm17_1130 were significantly overexpressed in ccRCC relative to nonmalignant cell lines, as shown by RT-qPCR (Supplemental Figure 3), which confirms the results observed from RNA-sequencing expression data. The expression of our newly detected sequences in cell lines not only confirms their presence in human kidney tissue but is also suggestive of their relevance to ccRCC biology. In light of these observations, further experiments may seek to elucidate the phenotypic consequences of previously unannotated miRNA deregulation.

Taken together, our results suggest that the discovery of previously unannotated miRNAs is an important next step in the exploration of the genomic landscape of tumourigenesis. While performed under the lens of kidney cancer, our findings have implications for cancers of all types, particularly in combination with previous studies that show these types of sequences to have a higher degree of specificity than those that are currently annotated. Although further validation and characterization of these types of sequences are required before these miRNAs can become clinically actionable, our findings highlight the untapped potential of the unexplored areas of the human transcriptome.

## 5. Conclusions

Here, we present an in-depth and large-scale discovery of previously unannotated miRNA sequences in human kidney samples. We find that not only are these sequences indeed expressed in both patient samples and cell lines but also their expression may be relevant to normal and tumour biology in these tissues. Several of these newly detected miRNAs are both deregulated in ccRCC tumours and associated with poor patient outcome. Moreover, protein-coding genes and subsequent pathways predicted to be targeted by these sequences, such as the VEGF and EGFR pathways, are critical to kidney tumourigenesis. Taken together, our results provide a novel resource for studying kidney cancer biology and underline a need for further identification of miRNAs that have eluded previous detection methods. Through the discovery of previously unannotated sequences, future studies may uncover novel players in tumour biology that may result in better characterization of ccRCC molecular drivers and direct new diagnostic and treatment strategies in the clinic.

## Data Availability

All data were accessed from the Genomic Data Commons platform through the National Institute of Health (https://gdc.cancer.gov/).

## Supplementary Materials

Supplementary 1 Figure 1: genome-wide distribution of previously unannotated miRNA sequences. Purple bars represent log_2_ (total read counts) for previously unannotated miRNAs detected in ccRCC samples, while green bars indicate those detected in nonmalignant tissues. The outer circle represents all chromosomes from the human genome.

Supplementary 2 Figure 2: unsupervised hierarchical clustering analysis of differentially expressed previously unannotated miRNA sequences. Both samples and features were clustered using average linkage and Pearson correlation. Standardized expression is represented shown from high (red) to low (green), and samples are coloured according to their malignant status (blue represents nonmalignant and red represents tumour tissue).

Supplementary 3 Figure 3: fold change (RQ) values from RT-qPCR analysis of Knm3_1968 and Knm17_1130 in TK-10 ccRCC cells relative to nonmalignant HEK-293T cells.

Supplementary 4 Table 1: summary of OASIS output and subsequent filtering steps of predicted novel miRNAs detected in kidney tissues. This table lists the raw output of the computational analysis deducing previously undetected miRNAs from RNA-sequencing data of nonmalignant (NM) and clear cell renal cell carcinoma samples (TP) (columns A–E) and the results from further filtering steps (columns F–J) as described in the “Materials and Methods.” Table 2: cell line characteristics and next-generation sequencing information for the NCI-60 renal cell lines used to validate the discovery of previously unannotated miRNA sequences. Table 3: predicted target genes of the newly detected miRNAs differentially expressed in clear cell renal cell carcinoma. This table lists the output values of the miRanda target prediction algorithm used to predict the protein-coding genes targeted by previously undetected miRNAs in kidney samples, as described in “Materials and Methods.”
